# Genetic loci for ventricular dilatation in the LEW/Jms rat with fetal-onset hydrocephalus are influenced by gender and genetic background

**DOI:** 10.1186/1743-8454-2-2

**Published:** 2005-06-12

**Authors:** Hazel C Jones, Crystal F Totten, David A Mayorga, Mei Yue, Barbara J Carter

**Affiliations:** 1 Department of Pharmacology and Therapeutics, University of Florida, Gainesville, FL 32610, USA; 2 Dr. H. C. Jones, Gagle Brook House, Chesterton, Bicester, Oxon OX26 1UF, UK

## Abstract

**Background:**

The LEW/Jms rat strain has inherited hydrocephalus, with more males affected than females and an overall expression rate of 28%. This study aimed to determine chromosomal positions for genetic loci causing the hydrocephalus.

**Methods:**

An F_1 _backcross was made to the parental LEW/Jms strain from a cross with non-hydrocephalic Fischer 344 rats. BC_1_ rats were generated for two specific crosses: the first with a male LEW/Jms rat as parent and grandparent, [(F × L) × L], designated B group, and the second with a female LEW/Jms rat as the parent and grandparent [L × (L × F)], designated C group. All hydrocephalic and a similar number of non-hydrocephalic rats from these two groups were genotyped with microsatellite markers and the data was analyzed separately for each sex by MAPMAKER.

**Results:**

The frequency of hydrocephalus was not significantly different between the two groups (18.2 and 19.9 %), but there was a significant excess of males in the B group. The mean severity of hydrocephalus, measured as the ventricle-to-brain width ratio, was ranked as B group < C group < LEW/Jms. For the both rat groups, there were several chromosomes that showed possible regions with association between phenotype and genotype significant at the 5% or 1.0% level, but none of these had significant LOD scores. For the C group with a female LEW/Jms parent, there was a fully significant locus on Chr2 with a LOD score of 3.81 that was associated almost exclusively with male rats. Both groups showed possible linkage on Chr17 and the data combined produced a LOD score of 2.71, between suggestive and full significance. This locus was largely associated with male rats with a LEW/Jms male parent.

**Conclusion:**

Phenotypic expression of hydrocephalus in Lew/Jms, although not X-linked, has a strong male bias. One, and possibly two chromosomal regions are associated with the hydrocephalus.

## Introduction

Fetal hydrocephalus occurs in humans from causes such as intraventricular hemorrhage and intrauterine infections, but in other cases the cause cannot be identified with certainty. Epidemiological studies provide evidence that hydrocephalus has a genetic component [1-3], although only one inherited form, X-linked hydrocephalus, has been characterized at the molecular level [4]. Rodent hydrocephalus mutants have been known for many years [5] and a few mouse mutants have been genetically characterized [6-8]. The publication of the first DNA assembly for the rat has enabled the rat genome to be integrated with DNA sequences from other species [9]. It is now possible to identify homologous regions between rat and human or rat and mouse, and to place disease-related genes from the human or mouse on the rat genome. Additionally, the identification of candidate genes for specific traits in rats is possible through comparative mapping. The study of disease-related genes in the rat will lead to a better understanding of inherited conditions in humans.

The LEW/Jms rat was first described in 1983 as being derived from an inbred strain of Wistar-Lewis rats and as having lethal fetal-onset hydrocephalus with a frequency that varied between litters from 12 – 25% and sometimes higher [10]. A six-generation pedigree showed that about 25% of the breeding pairs did not produce hydrocephalic pups. The authors concluded that there was a Mendelian autosomal recessive mode of inheritance.

Seven rats of the strain were received at the University of Florida in year 2000 and their DNA was tested with 87 selected microsatellite markers. All but two of these markers were both homozygous and homogeneous indicating that the strain was almost completely inbred [11]. Since 2000 the strain has been maintained by brother-sister mating and almost all successful breeding pairs have produced hydrocephalic offspring. This suggests that hydrocephalus may not be a Mendelian recessive trait [11]. Severe hydrocephalus is evident soon after birth from a domed head, with death occurring soon after weaning. Therefore the strain is maintained by breeding from apparently non-hydrocephalic rats because pups with overt disease do not survive to reproduce. It was found, however, that some adult ex-breeding rats have a milder form of hydrocephalus. These rats, however, did not produce pups with an increased frequency for hydrocephalus, which would have been expected with direct transmission of the trait. The overall frequency of hydrocephalus among pups was 27.7%, with a significant excess of affected males. Crossing to another rat strain, Fischer 344, produced a small number of pups with hydrocephalus (3%). A backcross from the F_1 _progeny to the LEW/Jms strain produced hydrocephalic pups, also with an excess of males and a frequency of hydrocephalus of 18.8% [11]. The presence of affected pups in the F_1 _generation and the high frequency of affected BC_1 _pups suggest that the trait may be semi dominant and controlled by one or possibly two genetic loci. This study aimed to perform a genome-wide scan and QTL analysis on backcross progeny to identify chromosomal region(s) associated with the hydrocephalus. Using gender-specific crosses, the genotyping has revealed one and possibly two loci associated with hydrocephalus.

## Materials and Methods

### Animals

For all experiments the 'Principles of Laboratory Animal Care' (NIH publication no. 86-33, revised 1985) was followed. All rats were pathogen free at the start of the experiment and were housed for the duration of the experiment in a single room under conventional conditions. Pathogen monitoring was performed periodically. The LEW/Jms strain was donated by Dr. K. Sudoh, University of Tokyo, to H.C.J. at King's College, London, UK, in 1987. Between 1991 and 2000, they were housed at the University of Manchester Institute of Science and Technology, Manchester, UK (C. S. Bannister). Seven animals transferred to the University of Florida in 2000 were the founder rats for the current breeding colony and for this backcross experiment. The animals used in this study were selected at random from the four breeding lines described previously [11]. Inbred Fischer 344 rats were purchased from Harlan (Harlan F344/Hsd). This strain does not develop hydrocephalus and was used in a previous genetic analysis with the H-Tx hydrocephalic strain [12].

### Breeding

In the first part of the study, the LEW/Jms (L) rats were bred to Fischer 344 (F) and the F_1 _progeny backcrossed to LEW/Jms as described previously producing 1574 backcross (BC_1_) progeny [11]. A genotype analysis using the complete set of BC_1 _progeny did not produce meaningful results. Hence the results were examined according to the sex of the parents (see Results, Analysis of genotypes). Of 1574, 599 had LEW/Jms as the paternal parent for both generations, designated 'B' group [(F × L) × L], 114 of which had hydrocephalus. A further 365 were designated 'C' group with LEW/Jms as the maternal parent for both generations [L × (L × F)] and 68 had hydrocephalus. Additional backcross breeding was carried out to increase the number of rats within these two specific crosses. For the 'B' group progeny, 8 female F_1 _rats bred from LEW/Jms males and F344 females, were crossed with 8 male LEW/Jms rats and 373 BC_1 _progeny were generated. For the 'C' group progeny, 24 male F_1 _rats bred from F344 males and LEW/Jms females, were crossed with 24 LEW/Jms females and 608 BC_1 _progeny were generated. Records of the breeding pairs and litters born were entered into a database (Filemaker Pro, Filemaker Inc, CA, USA) for both LEW/Jms breeding colony and the backcross.

### Analysis of phenotype

In a previous study it was shown that the severity of hydrocephalus as measured by the ratio of ventricle-to-brain width is independent of age between pups aged 2–23 days after birth. This was true for both the LEW/Jms colony and the BC_1 _rats [11]. To measure phenotype rats were euthanized with CO_2 _suffocation, or in the case of pups less than 10 days old, with an overdose of sodium pentobarbital (100 mg/kg). The brains were excised, fixed in 10% neutral buffered formalin and sliced coronally at 1 mm thickness using a fine blade. The slices were examined and photographed under a binocular microscope. A slice at the level of the striatum was photographed and the dilatation measured as the ratio of ventricle width-to-brain width (hydrocephalus severity, [13]). Non-hydrocephalic (control) rats were given a nominal phenotype of 0.01 because although ventricles are not visible on 1 mm slices, small ventricles are found in histological sections [14]. Phenotype measurements were entered into the MAPMAKER program for analysis. The data was compared to data from the brains of 392 rats from the LEW/Jms parental strain described previously [11].

### Genotyping

With the exceptions described below, BC_1 _progeny were sacrificed between 2 and 23 days of age. Liver tissue was removed, frozen in liquid N_2 _and stored at -80°C for DNA extraction. Because it was found previously that a proportion of ex-breeding LEW/Jms rats had ventricular dilatation when examined *post mortem *[11], some BC_1 _rats were raised until 22–24 weeks of age, at which stage they were sacrificed and tissues removed; n = 54 rats from five litters for the B group and n = 60 rats from five litters for the C group. Genomic DNA was extracted from liver tissue using the standard chloroform-phenol method, amplified by PCR using primers for microsatellite markers (Rat Map Pairs, Research Genetics or Invitrogen) and separated by agarose gel electrophoresis as described previously [15].

For the initial study, DNA was extracted from all 247 hydrocephalic rats and from 168 littermates that had no ventricular dilatation (non-hydrocephalic rats). This set of BC_1 _rats had mixed parentage, consisting of 29% that had a female LEW/Jms parent for the first generation and a male for the backcross, 39% with both LEW/Jms parents being male rats (designated 'B' type), 23% with a female LEW/Jms parent in the first cross and also in the backcross (designated 'C' type), and 8.5% with a female parent in the first cross and a male for the backcross. Genotyping was performed in stages using a panel of 96 genome-wide microsatellite markers. The mean spacing between markers was 14.36 cM and the largest was 41.64 cM on Chr20, where informative markers were scarce. Apart from this, two other markers on Chrs7 and 18 had spacings >30 cM (30.40 and 31.75) and all other spacings were < 30 cM. The results were examined with the χ^2 ^test for significant departure from the null hypothesis that the ratio of homozygous to heterozygous genotypes was 50:50.

For the additional rats generated in the B and C categories, DNA was extracted from the frozen liver of all overtly hydrocephalic pups an equal number of non-hydrocephalic littermates, and from pups found to have mild hydrocephalus after examination of the brains. First-stage genotyping was performed using DNA from 30 or more hydrocephalic and non-hydrocephalic rats from each group, with the same genome-wide panel of 96 microsatellite markers. The data was combined with data from the initial study, analyzed separately for each group, and examined for significance at the 5% level. The presence of significance at the 5% level, while not sufficient for likely linkage, was used as a guideline to determine the strategy for possible further genotyping. Genotyping was then continued on specific chromosomes where there was significant association, until all rats had been included. Additional markers were included to increase the density on these chromosomes. The data for each chromosome was analyzed by MAPMAKER.EXP to determine the best marker order and by MAPMAKER.QTL to calculate the LOD score [16]. Significance levels were determined using a LOD score of 1.9 (*P *< 0.0034) for suggestive significance and a score of 3.3 (*P *<0 .0001) for full significance as defined by Lander & Krugylak [17].

### X Chromosome analysis

Since there was an excess of males with hydrocephalus, a possible association with ChrX was sought. Genotypes of the B type progeny with a male LEW/Jms parent at each generation, were informative for ChrX. The C progeny could not be used because the contribution from the non-hydrocephalic F344 strain came from ChrY. Seven microsatellite markers polymorphic for the LEW/Jms × F344 cross were genotyped on all 'B' rats and the data analyzed independently for each sex by the χ^2 ^test.

### Human-rat homology to search for candidate genes

Candidate genes were sought for the chromosomal regions where there was significant evidence for a hydrocephalus locus as determined by MAPMAKER. A strategy was used similar to that described previously for the H-Tx rat linkage analysis [12]. The Ensemble Rat Genome Browser, a joint project between the European Molecular Biology Laboratory-European Bioinformatics Institute and the Sanger Institute (, version 26.3 d.1, 08/02/2004) was used to identify the megabase (Mb) positions for the microsatellite markers in the vicinity of the hydrocephalus loci. The likely genetic positions for the loci were identified based on the LOD score maps generated by MAPMAKER. Possible candidate genes were selected from known rat genes and from predicted genes that were identified from homologous regions on the human and the mouse genomes. Genes were then evaluated as potential candidates using a number of different criteria [12].

## Results

### Breeding and expression of hydrocephalus

**Figure 1:**
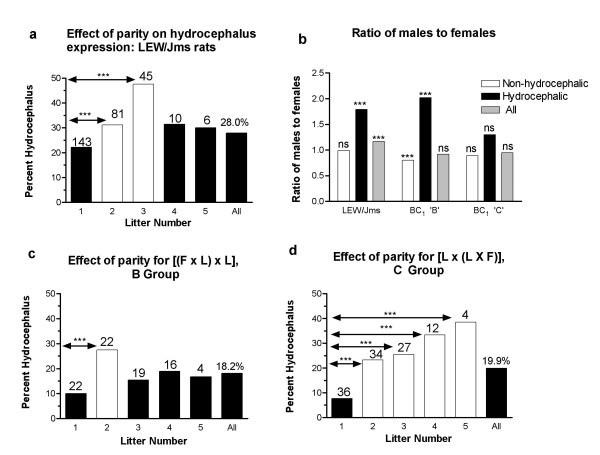
a: Percentage of hydrocephalus in LEW/Jms rats by litter number (parity) and total percentage (All). There was a significant increase in percentage of affected rats between the 1^st ^and 2^nd ^and the 1^st ^and 3^rd ^litters, *P *< 0.001. This was not maintained for the 4^th ^and 5^th ^litters. The number of litters in the data sets is depicted above the columns.b: The ratio of males to females in the parental LEW/Jms strain and in the BC_1 _progeny. In the parental strain, there were significantly more males than females with hydrocephalus (black bars, *P *< 0.001), and also among the total pups born (striped bars). In the B group [(F × L) × L], there was a significant excess of females among the non-hydrocephalic pups, (open bars, *P *< 0.001 and a significant excess of males in the hydrocephalic pups, *P *< 0.001. In the C group [L × (L × F)], there were no significant departures from the expected 1:1 sex ratios for the hydrocephalic or the non-hydrocephalic rats (ns).c: Percentage of hydrocephalus in [(F × L) × L] BC_1 _rats by litter number (parity) and in total. There was a significant increase in the percentage expressing hydrocephalus between the 1^st ^and 2^nd ^litters, *P *< 0.001. This was not maintained in subsequent litters. The number of litters in the data sets is depicted above the columns. d: Percentage of hydrocephalus in [L × (L × F)] BC_1 _rats by litter number (parity) and in total. There was a steady and significant increase in expression between 1^st ^litters and all subsequent litters, *P *< 0.001. The number of litters in the data sets is depicted above the columns.

LEW/Jms parental strain: From breeding records kept over a period of 3.5 y, the overall frequency of overt hydrocephalus was 28% out of a total of 2401 pups (Fig. 1a, column 'all'). As reported previously, there were significantly more males with hydrocephalus than females, χ^2 ^= 46.21, but no significant difference between the sexes of non-hydrocephalic rats. Instead there was a small, but significant excess of total males over total females, χ^2 ^= 12.3 (Fig 1b). The frequency of hydrocephalus varied with parity, in that the percentage in second and third litters, 31.2% and 47.6% respectively, was significantly increased over that in first litters, 22.2%, *P *< 0.001, χ^2 ^test (Fig 1a). Although the frequency in the fourth and fifth litters was also increased over the first litter, there was no statistical significance, possibly because the numbers were small as some females did not continue to breed after the third litter. The average litter size decreased from 9.5 pups in first litters to 5.0 in fifth litters.

### Backcross progeny

Table 1 shows the total number of BC_1 _pups sacrificed at 2–23 days of age and numbers genotyped for each group. There was an excess of males over females with hydrocephalus in the B group, χ^2 ^= 15.57 (Table 1, Fig. 1b). The same trend was also present in the C group, but not significant. Both groups also had an excess of female over male non-hydrocephalic rats, although again, it was only significant for the B group, χ^2 ^= 11.02. The frequency of hydrocephalus in the B and C backcross progeny was not significantly different between the two groups, 18.2% and 19.9%, respectively (Figs. 1c and 1d, column 'All'). Similar to the parental LEW/Jms strain, the frequency of hydrocephalic pups depended on parity and was significantly lower in the first litters than in subsequent litters. This effect was much less evident for the B progeny than for C progeny, which had the female LEW/Jms parent (Figs. 1c and 1d). The ratio of male to female hydrocephalic pups was not significantly different between the 1^st^, 2^nd^, 3^rd ^and 4^th ^litters for either group, χ^2 ^test. Among the backcross progeny sacrificed at 22–24 weeks, there were seven rats with mild hydrocephalus in the B group (n = 54) and nine in the C group (n = 60). An additional two had severe, but non-fatal disease in the B group and three in the C group. As with the pups, there was also an excess of males with hydrocephalus in these two groups. However, the sample was small and there was no statistical significance.

**Table 1:** Numbers of BC_1 _pups of each phenotype bred and genotyped (in parenthesis). In the B group there were more males than females with hydrocephalus, *P *< 0.001 (a) and more females than males in the non-hydrocephalic group *P *< 0.001 (b). The differences were not significant for the C group.

	Parentage	Sex	Hydrocephalic	Non-hydrocephalic	Total
B Group	[(F × L) × L]	Male	109 (108)^a^	330 (91)	439 (198)
		Female	58 (58)	421 (51)^b^	479 (109)
C Group	[L × (L × F)]	Male	102 (102)	342 (82)	444 (184)
		Female	80 (79)	386 (58)	466 (137)

### Analysis of Phenotypes

The mean severity of ventricular dilatation for the B group was 0.58 +/- 0.01 and for the C group was 0.61 +/- 0.01. This difference was significant, *P *< 0.05, Kruskal-Wallis test. There was no significant difference between the mean severity for males and females in either group (data not shown). Dilatation severity was 0.66 +/- 0.01 for the LEW/Jms parental strain, which was significantly higher than for B or C rats, *P *< 0.01 and 0.05, respectively. As reported previously, there was no significant difference in hydrocephalus severity between males and females in the parental strain [11].

### Analysis of genotypes

The genotypes for the first backcross progeny with rats from mixed mating groups were examined using χ^2 ^test for association between phenotype and genotype. On Chr17 at marker D17Rat17, there was significance at the level of *P *< 0.05. No other marker on any chromosome had a significant result. The data was re-tested after separation of the genotypes into four groups according to sex of the parental rats. However, as already stated, the number of rats was too low at this stage for the results to be meaningful. The genotypes obtained for B and C rats in the initial study were combined with data for the additional B and C rats bred subsequently. First, the genotypes were analyzed for the male and female data combined. In addition, because of the strong male bias in the expression of hydrocephalus, the data was analyzed separately for males and for females (Tables 2 and 3).

**Table 2:** Genotypes for B type [(F × L) × L] male and female rats showing markers for which the association between phenotype and genotype was significant at a level of 0.05 (*) or 0.01 (**).

[(F × L) × L] Males		Genotypes					
				
		Hydrocephalic		Non-hydro-cephalic			
		LL	LF	LL	LF	χ^2^	*P *value
**Chr1**	D01Rat56	45	63	51	40	**4.09**	*
							
	D01Rat57	46	62	43	27	**6.03**	*
							
	D01Rat65	47	60	52	37	**4.09**	*
							
	D01Rat219	40	60	52	39	**5.61**	*
							
	D01Rat67	47	59	54	38	**4.06**	*
							
	D01Rat208	46	62	52	39	**4.18**	*
							
**Chr 5**	D05Rat49	45	33	26	38	**4.10**	*
							
**Chr 11**	D11Rat28	50	42	32	54	**5.25**	*
	D11Rat73	53	40	35	56	**6.33**	*
							
**Chr17**	D17Rat85	65	40	40	51	**6.31**	*
	D17Rat65	68	39	40	50	**7.21**	**
							
**Chr19**	D19Rat28	68	40	43	52	**6.39**	*
	D19Rat12	66	41	42	52	**5.82**	*
	D19Rat40	68	39	44	45	**3.95**	*
	D19Rat95	63	43	32	41	**4.22**	*
							
		L	F	L	F	χ^2^	*P *value
**ChrX**	DXRat83	60	43	39	51	**4.28**	*
							
(F × L) × L] Females		LL	LF	LL	LF	χ^2^	*P *value

**Chr1**	D01Rat36	24	34	31	19	**4.57**	*
							
**Chr5**	D05Rat36	35	23	19	32	**5.79**	*
	D05Rat41	38	18	24	27	**4.74**	*
							
**Chr13**	D13Rat85	19	28	32	18	**5.40**	*
							
**Chr19**	D19Rat28	36	24	22	33	**4.59**	*

**Table 3:** Genotypes for C type [L × (L × F)] male and female rats showing markers for which the association between phenotype and genotype was significant at a level of 0.05 (*) or 0.01 (**).

		Genotype					
				
[L × (L × F)] Males		Hydrocephalic		Non-hydro-cephalic			
		LL	LF	LL	LF	χ^2^	*P *value
**Chr2**	D02Rat15	61	40	34	45	**5.36**	*
	D02Rat34	63	41	34	48	**6.71**	**
	D02Rat91	60	37	36	43	**4.66**	*
	D02Rat52	64	40	37	45	**4.98**	*
	D02Rat49	68	37	38	42	**5.53**	*
	D02Rat46	66	38	39	41	**3.99**	*
	D02Rat241	67	31	35	43	**9.84**	**
	D02Rat62	65	40	34	48	**7.72**	**
	D02Rat65	59	19	39	31	**6.55**	*
	D02Rat250	58	38	32	48	**7.28**	**
							
**Chr4**	D04Rat112	37	66	47	34	**8.93**	**
	D04Rat72	41	62	46	34	**5.65**	*
							
**Chr10**	D10Rat28	36	10	25	22	**6.47**	*
							
**Chr16**	D16Rat81	32	53	32	25	**4.71**	*
							
**Chr17**	D17Arb3	66	42	38	47	**5.15**	*
	D17Arb13	66	41	38	47	**5.50**	*
	D17Rat181	63	40	37	44	**4.38**	*
	D17Rat17	66	40	39	45	**4.75**	*
							
[L × (L × F)] Females		LL	LF	LL	LF	χ^2^	*P *value

**Chr2**	D02Rat21	48	25	23	35	**8.87**	**
							
	D02Rat135	47	28	21	36	**8.65**	**
	D02Rat26	48	25	25	28	**4.35**	*
	D02Rat34	46	30	23	35	**5.74**	*
	D02Rat91	49	25	23	35	**9.25**	**
	D02Rat46	48	26	21	35	**9.58**	**
	D02Rat52	52	24	26	32	**7.53**	**
	D02Rat49	51	24	26	32	**7.20**	**
							
**Chr7**	D07Rat195	19	27	28	14	**5.68**	*
							
**Chr17**	D17Arb4	32	25	21	36	**4.27**	*
	D17Rat181	41	34	21	36	**4.13**	*
	D17Rat17	43	32	22	35	**4.55**	*
	D17Arb5	43	27	25	33	**4.28**	*

#### B group QTL mapping

**Figure 2:**
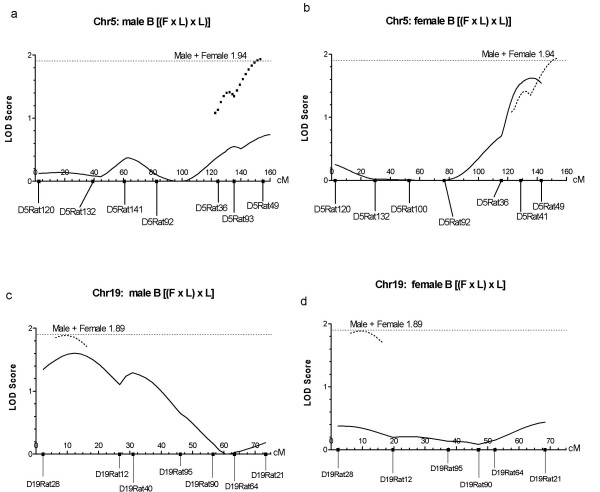
LOD score graphs created from the MAPMAKER output for [(F × L) × L], B rats on chromosomes 5 and 19. The X-axes represent the recombination distance in centi-Morgans (cM). The microsatellite markers are positioned on the X-axis and named below. The horizontal dotted line represents the score required for suggestive significance (1.9). a: Plot for male B rats on Chr 5 and b: plot for female B rats (solid lines), dotted line represents a portion of the plot for both sexes combined, where the LOD score (1.94) reached suggestive significance. There was a strong female bias (3b). c: Plot for male B rats on Chr 19 and d: plot for female rats (solid lines). The dotted line near the centromeric end represents part of the plot for both sexes combined where the LOD score (1.89) is very close to suggestive significance for linkage. The male rats contributed to this peak almost exclusively.

Analysis of the data for male and female rats combined showed two or more markers on each of four chromosomes with significance at the 5% level or above by χ^2 ^test (Chrs 1, 5, 17, and 19). QTL analysis was performed with MAPMAKER for these chromosomes using the combined data, and also on data for each sex separately. None of the chromosomes reached the level required for full significance (3.3) for both sexes combined, but a score suggestive for significance was achieved on Chr5, LOD = 1.94, and almost achieved on 19, LOD = 1.89 (Fig 2). Separate male and female MAPMAKER analyses for chromosome 5 indicated a greater level of significance for females than males (Table 2, Figs. 2a, 2b). For chromosome 19, there was a higher significance level for the males (Table 2, Fig. 2c) and the contribution of the females to the combined LOD score was very small (Fig. 2d). On Chr17, the peak LOD for both sexes was 1.84 at D17Rat65 and again, the effect was largely on the males (Table 2). The X chromosome marker DXRat83 gave a significant result, P < 0.05 for the male rats in this B group (Table 2). In addition, the data showed that chromosomes 11 (for males) and 13 (for females) had results for one or two markers significant at the 5% level or higher (Table 2). The low significance levels and small LOD scores obtained for this group of rats did not contribute meaningful results to this genetic analysis. The exception was data for Chr17, where the results were combined for the B and C groups (see below).

#### C group QTL mapping

**Figure 3:**
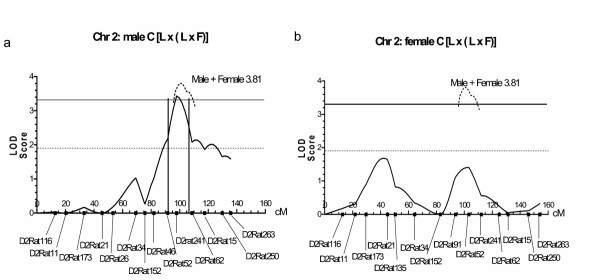
LOD score graphs created from the MAPMAKER output for [L × (L × F)] C rats on Chr 2. The X-axes represent the recombination distance in centi-Morgans (cM). a: plot for male rats and b: for female rats (solid line). The horizontal dotted line represents the score required for suggestive significance (1.9) and the solid line the score for full significance (3.3). The two graphs are quite different for males and females with the males reaching a score indicative of full significance (3.43) in the same location as the map for both sexes combined (dotted line, LOD = 3.81).

The combined analysis for male and female C rats showed quite different results to that seen for the B group. Instead, chromosomes 2 and 4 were significant for two or more markers at the 5% level or above. Of these, chromosome 2 had a peak of LOD = 3.81, indicating a locus with full significance for hydrocephalus situated near D2Rat241 (Figs 3a,b). The LOD score for chromosome 4 (1.59) did not reach the level for suggestive significance. Similar to the B group, there was a peak on Chr17 at a different location, D17Rat13, LOD = 1.73, but it was not significant. The males and females were analyzed separately (Table 3). The locus on Chr2 was gender specific in that it had a much larger effect on males with a LOD score of 3.43, whereas for the females the LOD score was only 1.41 (Table 3, Figs. 3a,b). The effect on Chr4 was almost totally on the male rats (Table 3). For Chr17, males and females were affected equally (Table 3). In addition to chromosomes 2, 4, and 17, described above, chromosomes 10 and 16 (for males) and 7 (for females) had data for one or more markers significant at the 5% level or above (Table 3). Only Chr2 was studied further, apart from Chr17 where the data was combined for both groups (see below).

**Figure 4:**
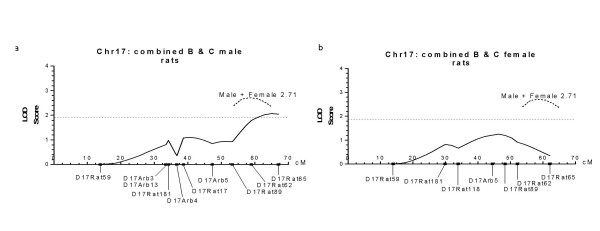
LOD score graphs created from the MAPMAKER output for Chr 17, using the combined data for both B and C rats. The X-axes represent the recombination distance in centi-Morgans (cM). The horizontal dotted line represents the score required for suggestive significance (1.9). a: plot for male rats and b: for female rats (solid lines). The combined male and female score represented a locus that was between suggestive and full significance (dotted plot, LOD = 2.71). This locus also had a male specificity that on further analysis was shown to come from the male B rats (data not shown).

#### B and C QTL combined

All data sets were combined for QTL analysis of Chr17. The maximum LOD score for all rats was 2.71, between suggestive and full significance, and situated near D17Rat62. For males it was 2.07, also close to D17Rat62, but for females the maximum LOD was only 1.26 and situated in a different position at D17Arb5 (Figs. 4a,b). Hence this may be a second gender-specific locus. The male bias largely came from the B group with the male LEW/Jms parent as described above.

In summary, the genotype analysis of B rats with a male LEW/Jms parent and grandparent showed no chromosomal regions indicative of a locus for hydrocephalus, with the possible exception of Chr17. On the other hand, analysis using C rats with a female LEW/Jms parent and grandparent showed a locus with significant linkage for hydrocephalus on Chr2 that chiefly affected males. The locus on rat Chr2 is situated at 217–218 Mb in band q41. The region at and around the locus is homologous to human chromosomes 1 and 4. Chromosome 17 linkage was common to both rat groups. There was a region of Chr17 that was between the suggestive and fully significant level for hydrocephalus when the data was combined. In this case, the locus acted on both sexes but more so with the male rats from the B group. The peak was situated close to D17Rat62 located at 83.5 Mb in band 17q12.3. This region is homologous to human 10p14 at 12.25 Mb.

## Discussion

The LEW/Jms rat is a model for fetal-onset human hydrocephalus. In the human, ventriculomegaly, defined as dilated lateral ventricle atria, can be detected by ultrasound examination from 20 weeks of gestation and sometimes earlier [18]. In some cases the dilatation remains stable or resolves. In other cases there is progression to hydrocephalus with increased head circumference and a requirement for shunt treatment in the post-natal period. Fetal hydrocephalus is frequently associated with a poor neurodevelopmental outcome [18,19]. In many cases the primary cause is uncertain, but stenosis of the cerebral aqueduct is often a feature [20]. The LEW/Jms rat model falls into this category, having fetal-onset progressive hydrocephalus with an abnormal aqueduct [21,22]. In many respects the phenotype is similar to hydrocephalus in the H-Tx strain [23,24]. Both strains have severe fetal-onset disease associated with aqueduct stenosis and dysplasia of the subcommissural organ [14,21,22]. Hydrocephalus expression in H-Tx rats has been shown to be polygenic and influenced by at least four loci on different chromosomes [12] and by strong epigenetic effects [25]. However, neither gender nor cross-specific effects were observed in H-Tx. A surprising observation reported in this study was the increase in the frequency with parity from 22.2% in first litters to 47.6% in third litters. This appears to be a similar phenomenon to that observed in the H-Tx rat, where it was found that the frequency of hydrocephalus was lower in first litters than in subsequent litters [25]. In H-Tx hydrocephalus, the increase in hydrocephalus frequency among the pups *in utero *was associated with concurrent suckling by the dam of a previous litter. In the case of LEW/Jms rats, there was a progressive increase in frequency with parity but whether or not the phenomenon was related to concurrent suckling was not investigated. It does indicate, however, that there may be epigenetic effects affecting the expression of hydrocephalus in this strain as occurs in the H-Tx strain.

The results of this study suggest that there is a locus for hydrocephalus on Chr2, as shown in male rats with a female LEW/Jms parent. There is possibly a second locus on Chr17 that is associated with hydrocephalus in rats with parents of either sex, although the males with a male LEW/Jms parent made the largest contribution. This is the first time that a genetic analysis has been attempted in the LEW/Jms rat strain. It was reported previously that twice as many male as females rats are affected with hydrocephalus [11]. Although DNA samples from the BC_1 _progeny with a male LEW/Jms parent were tested with seven ChrX markers, only one marker, DXRat83 at position 43.2 Mb in band q21 on the X chromosome (Ensembl Rat Genome Browser ), showed a low level of significance (*P *< 0.05) with the male rats. It therefore seems unlikely that X chromosome linkage is involved despite the fact that X-linked hydrocephalus is well characterized in humans [4] and is due to mutations in the gene coding for L1 neural cell adhesion molecule. A more likely explanation for the specific sex effects is that gender affects phenotypic expression in this strain. Gender-specific loci have been observed in the analysis of other quantitative traits in rodents [26-28]. One explanation for this phenomenon is that genes on the autosomes, such as those coding for sex hormones, may influence the expression of disease-related genes.

**Figure 5:**
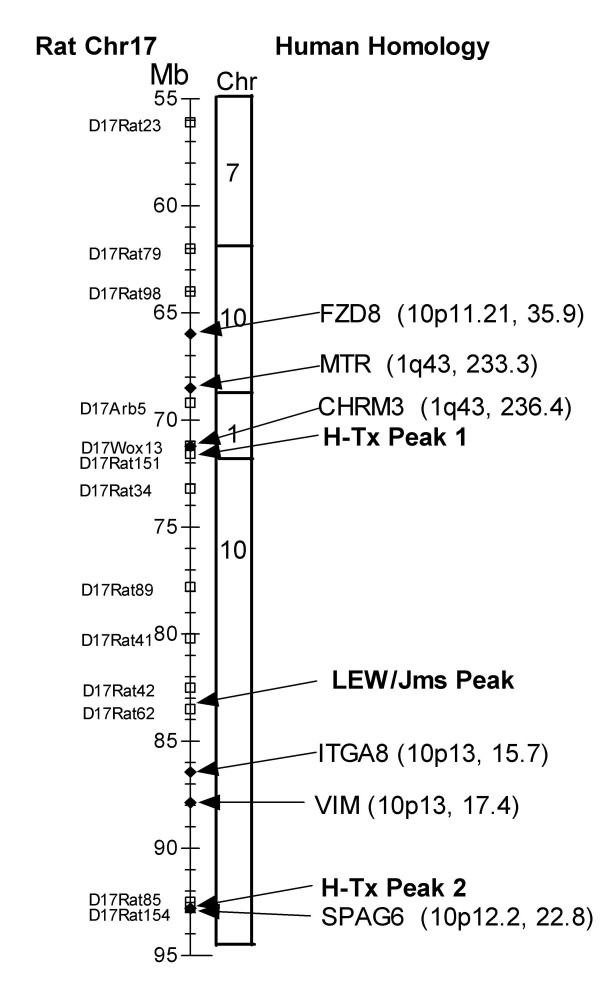
A schematic map for the hydrocephalus locus on rat Chr17. The scale on the left is the rat genetic length in megabases (Mb). Positions for the rat DNA markers (open squares) were identified from Rat Genome Browser, Ensemble web site . To the right of the rat chromosome are the human chromosome homologues. The positions for two possible hydrocephalus loci found in a previous study on H-Tx rats [12], and the peak for LEW/Jms rats in this study are marked (arrows). Possible candidate genes (black diamonds and arrows) are named and their human cytogenetic and Mb positions given in parenthesis.

In a recent genetic analysis of inherited hydrocephalus in the H-Tx rat, a region of Chr17 was identified at 25 – 55 cM that was highly significant for hydrocephalus in this strain. It was concluded that one or more loci exist in this region [12]. The equivalent genetic map positions were 71.2 – 92.6 Mb. This large region is homologous to human Chr1q43 and 10p11.21-p13. The locus identified here for LEW/Jms rats is located in the middle of this section, close to D17Rat62 located at 83.5 Mb in band 17q12.3. This region is homologous to human 10p14 at 12.25 Mb (Fig. 5). It is possible that the two strains share a common susceptibility locus for hydrocephalus on Chr17. There are three possible candidate genes in this region close to D17Rat 62. One is SPAG6 at human 10p12.2 and 22.6 Mb. This gene codes for sperm-associated antigen isoform 1 which is the murine homologue of a component of the central flagella apparatus in sperm flagellae. Spag6 knockout mice are infertile and have hydrocephalus [29]. These mice may have impaired cilia function in the brain, a potential cause for hydrocephalus [30,31]. Two other known genes located within 10 Mb of the locus are ITGA8 or integrin alpha 8 at human 10p13 and located at 15.6 Mb, and VIM coding for vimentin at human 10p13 and located at 17.3 Mb. Both of these genes are important for brain development [32,33] but have no known association with hydrocephalus. Three more candidate genes are located on this chromosome close to the largest linkage peak for the H-Tx strain but further from the LEW/Jms peak [12]. These are FDZ8, frizzled 8 precursor which codes for a Wnt receptor, MTR, methyltetrahydrofolate-homocysteine methyltransferase or vitamin B_12_-dependant methionine synthase, and the gene for the acetylcholine muscarinic type 3 receptor, CHRM3.

**Figure 6:**
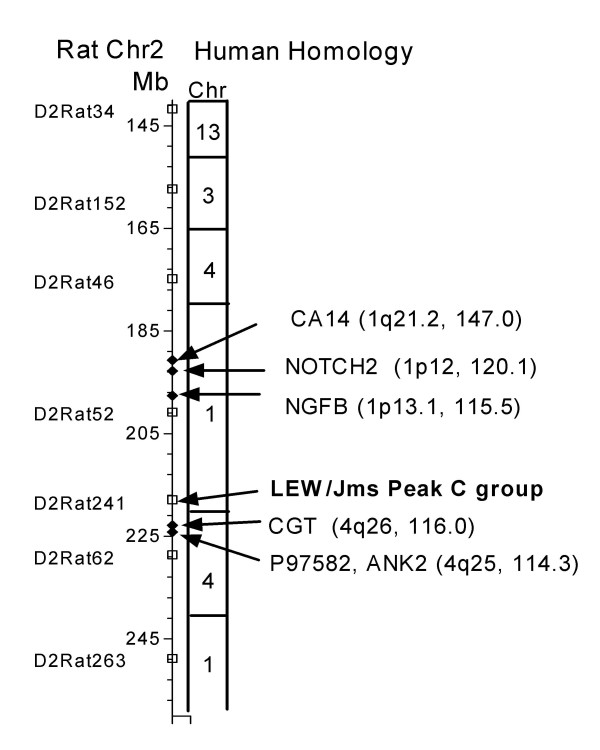
A schematic map for the hydrocephalus locus on rat Chr2. The scale on the left is the rat genetic length in megabases (Mb). Positions for the rat DNA markers (open squares) were identified from Rat Genome Browser, Ensemble web site . To the right of the rat chromosome are the human chromosome homologues. The peak LOD score for LEW/Jms rats is marked (arrows). Possible candidate genes (black diamonds and arrows) are named and their human cytogenetic and Mb positions given in parenthesis.

The locus identified by MAPMAKER on Chr2 was located close to marker D2Rat241 at 217–218 Mb in band q41. This region is homologous with human Chr1 and with a section of human Chr4 (Fig 6). It is also homologous with mouse Chr3 and includes a region that is particularly rich in genes that are transcribed in the nervous system [34]. The regions at and around the locus were examined for possible candidate genes using Ensemble Genome Browser to identify rat genes and homologous human or mouse genes that are expressed in brain and might have an association with hydrocephalus. One such gene is P97582 (rat 224.2 Mb) or ANK2 (human Chr4, 114.3 Mb), which codes for brain ankyrin or ankyrin_B_. Ankyrins are spectrin-binding proteins on cell membranes that associate with L1 CAM, and with several ion channels. Ankyrin_B _deficient mice have a similar phenotype to L1 deficient mice with features that include dilated cerebral ventricles [35]. Close by at 222.9 Mb is CGT or 2 hydroxyacyl sphingosine 1-B-galactosyltransferase, a gene found in oligodendrocytes and involved in myelination [36]. Another attractive candidate is NGFB, or beta nerve growth factor precursor at position 197.2 Mb. NGF and other neurotrophins and their receptors are upregulated in brain damage including that caused by hydrocephalus [37]. Furthermore, hydrocephalic H-Tx rats have alterations in brain NGF concentrations [38] and children with hydrocephalus have elevated NGF in the CSF [39]. NOTCH2, or notch homologue protein 2 precursor, is at 192.8 Mb and close to NGFB. Notch proteins are transmembrane receptors involved in cell fate determination in the CNS and Notch2 is important for roof plate development [40]. Notch2 affects Wnt-1 expression, and in mouse the Wnt sw/sw mutant has defective SCO development and hydrocephalus [41]. Also in the same region of Chr2 is CA14 or membrane-associated carbonic anhydrase XIV precursor at 190.65 Mb. This isoform of carbonic anhydrase is expressed in choroid plexus in addition to neuronal cells but its function is not clear [42] although another isoform CAII plays an important role in CSF secretion at the choroid plexus [43].

The identification of possible candidate genes is extremely speculative because of the low resolution obtained from QTL linkage analysis and the fact that the chromosomal regions identified contain many hundreds of genes. In contrast to genetic diseases with Mendelian inheritance, QTL mapping for complex traits has not, in most cases, led to the identification of abnormal genes [44]. However, additional strategies are available such as expression profiling in disease states, DNA sequencing for polymorphisms in candidate genes and transgenic technology all of which can lead to gene identification. In conclusion, the genetic basis for hydrocephalus expression in LEW/Jms rats is associated with one or possibly two genetic loci and in addition, the phenotypic expression is strongly influenced by gender.

## Competing interests

The author(s) declare that they have no competing interests.

## Authors' contributions

HCJ conceived of the study, was responsible for its design and coordination and writing the manuscript. DAM and BJC participated in rat breeding. CFT, DAM, MY and BJC all participated in phenotype and genotype analysis. CFT was responsible for the male/female analysis, and assisted in figure, table and manuscript preparation.
